# Penile Flap Inversion Vaginoplasty in Transgender Women: Contemporary Morbidity and Learning-Curve Analysis From a High-Volume Reconstructive Center

**DOI:** 10.3389/fsurg.2022.836335

**Published:** 2022-02-23

**Authors:** Valentin Maurer, Marian Howaldt, Inga Feldmann, Tim Ludwig, Malte W. Vetterlein, Philipp Gild, Sylvia Weis, Phillip Marks, Armin Soave, Christian P. Meyer, Margit Fisch, Roland Dahlem, Silke Riechardt

**Affiliations:** Department of Urology, University Medical Center Hamburg-Eppendorf, Hamburg, Germany

**Keywords:** transgender, penile inversion vaginoplasty, learning curve analysis, transgender surgery, reconstructive urology

## Abstract

**Objectives:**

Numbers of PIV are rising. The aim of this study is to analyze the surgical learning-curve (LC) on the grounds of perioperative complications.

**Patients and Methods:**

108 PIVs, performed by a single surgeon between 2015 and 2018 have been analyzed. Learning-curve analysis was based on three factors: operating time, vaginal depth and complications.

**Results:**

The median FU was 6.3 months. Median age at surgery was 36 years, median time of hormone treatment was 36 months. The median CI was 0.3 and the median BMI was 25 kg/cm3. Median CCI® was 8.08. 40.7% of the patients developed short-term complications, more than half of which were Clavien I. Overall only 1.9% had Clavien IIIb complications. There were no Clavien IV or V complications. 17.6% of patients had wound infections, 13% wound dehiscence, 9.3% introitus strictures, 13.9% suffered from atrophy of the neovagina, i.e. loss of depth or width, and 8.3% from meatus urethrae strictures. Duration of hormonal therapy, BMI and CI had no impact on surgical outcome. Age had a significant impact on CCI®, as younger patients had a higher risk for complications. Use of scrotal skin and surgeries performed had a significant influence. LC analysis *via* CUSUM analysis showed that after 32 surgeries, the PIV is performed safely.

**Conclusion:**

The PIV is a safe GAS-technique, associated with minor complications leading to low rates of revision surgery. Younger age, the use of scrotal skin and surgeon's experience has significant impact on complications. Duration of hormonal therapy, circumcision and BMI has no impact on complications.

## Introduction

Over the last decade, gender-affirming surgery (GAS) has gained center stage, given that medical and surgical needs of patients presenting with GD are more frequently recognized (1, 2). In a recent granular systematic review of the literature from 2009 to 2019, estimates of self-reported “transgender” identity ranged from 0.3 to 2.7% (1). This is reflected by a rising number of

GAS procedures. Moreover, the importance of multidisciplinary care in this context has been recently elucidated by a systematic literature review showing that patients benefitted with regard to psychological well-being (3).

Despite the increasing relevance of transgender health, literature with respect to complications and learning-curve analyses in the context of GAS is scarce. Moreover, morbidity data are often not cohesive due to different surgical approaches, are limited by small study samples, and are commonly prone to underestimation due to a lack of standardized reporting methodology (4, 5). To the best of our knowledge, no complication analysis has been published using the Comprehensive Complication Index (CCI®) (6), which allows for an in-depth complication analysis, given that it mirrors the cumulative morbidity burden (5, 6) as opposed to only reporting the highest grade complication by the CDC (7).

The most common surgical approach for male-to-female GAS is the penoscrotal inversion vaginoplasty (PIV) (8–11). Potential clinical factors such as prior hormonal therapy, a history of circumcision, age, or BMI to predict perioperative complications following PIV are widely unknown. Additionally, data are scarce regarding a surgical learning-curve of PIV, and only few, smaller single-center studies have looked into this and suggested a minimum of 30 to 40 procedures to provide an environment of a safe and successful intervention (12, 13).

Against this backdrop, we firstly aimed to describe perioperative, short- and long-term complications and functional outcomes of PIV and to identify risk factors of perioperative complications. Secondly, we performed a contemporary PIV learning-curve analysis based on CUSUM to externally validate previously published data.

## Patients and Methods

### Study Population and Workflow

In accordance with an institutional review board approval, perioperative and FU-data of transgender women undergoing two-stage PIV at our multidisciplinary transgender center between 2015 and 2018 were retrospectively collected. Diagnostic workup and perioperative management were performed according to the World Professional Association for Transgender Health (WPATH) standards (14). All patients were seen in our outpatient clinic 6 weeks postoperatively for clinical check-up. Moreover, all patients were readmitted for the second procedure 6 months after the first stage.

### Surgical Procedure

All procedures were performed by one experienced reconstructive high-volume surgeon. The perioperative management was based on a standardized institutional protocol, which is in accordance with WPATH recommendations (14). Each patient received perioperative i.v. antibiotics (cefuroxime). Hormonal therapy was not paused. The first stage of PIV generally involves orchiectomy, penectomy, vaginoplasty, clitoroplasty, and labiaplasty. Patients are placed in a lithotomy position. Following orchiectomy, the scrotum is used to create the neolabia. Penile degloving is followed by the mobilization and occlusion of the prepuce. In case of a history of circumcision, a scrotal skin flap is used to augment the neovagina. A space between the spatulated urethra and the rectum is prepared *via* blunt preparation. The anticipated neovaginal depth is more than 14 cm. A spacer is placed in the neovagina and left *in situ* for at least seven days. The clitoris is fixated with its neurovascular bundle ventrally to the neovagina.

After the clinical check-up 6 weeks after surgery, patients are readmitted 6 months later for the second stage, which involves cosmetic corrections (i.e., skin reduction, enlargement of the introitus and tightening of the labia majora). If necessary, a simultaneous meatoplasty is performed.

### Follow-Up

All patients were admitted for at least 10 days and FU was performed according to our institutional protocol. Functional outcome was objectified by clinical examination, neovaginal stent placement and depth measurement. At the time of readmission for the second stage, a physical examination including neovaginal stent placement, depth measurement and uroflowmetry was performed.

### Covariables

We assessed preoperative clinical characteristics such as age, BMI, history of circumcision, comorbidities and duration of hormonal therapy. Additionally, we recorded surgical characteristics such as operative time and estimated blood loss. Complications were assessed according to the CDC (15) and the CCI® was calculated to evaluate cumulative morbidity (6).

Short-term (ST) complications including bleedings, infections, wound dehiscence, rectovaginal injury and deep vein thrombosis/ pulmonary embolism were defined as those occurring in the perioperative setting. Long-term (LT) complications including urethral necrosis, clitoral necrosis, introitus strictures, neovaginal atrophia, rectovaginal fistula and urethral fistulas were defined as those occurring in the follow-up observations at 6 weeks until readmission for the second stage 26 weeks later.

### Statistical Analyses

Firstly, we performed descriptive analyses of clinical and surgical covariables and perioperative complications. Means, standard deviations (SDs), frequencies and proportions were used for continuous and categorical variables, respectively.

Secondly, we employed multivariable linear regression with the CCI® as an endpoint to identify risk factors of a higher cumulative morbidity burden.

Thirdly, learning-curve analysis was performed using CUSUM analysis and two validated methods were employed to depict the longitudinal surgical learning based on operative time and cumulative perioperative morbidity (CCI®). In a scatterplot representation, a standard curve and a fractional polynomial prediction were drawn for the outcome measures (operative time, CCI®). Splitting method was used to arbitrarily divide the series in two equal groups. The means between the groups were statistically compared as a quality control. The learning-curve was further analyzed with the cumulative sum (CUSUM) to indicate the tipping point of no longer accumulating LT complications. CUSUM has been proven to be a valuable graphical method of quality control, showing objective evidence and changes in competence of a surgeon over time (16).

All analyses were performed using Stata® (StataCorp. 2013. Stata Statistical Software: Release 13. College Station, TX: StataCorp LP). Two-sided statistical significance was defined as a *p* < 0.05.

## Results

### Clinical and Surgical Characteristics

Of 108 transgender women, mean age at surgery was 36 ± 13 years, mean body mass index was 25 ± 6.1, mean CI was 0.3 ± 0.9, and 22 patients (20%) were treated for clinically diagnosed depression at the time of surgery. Mean duration of prior hormonal therapy was 3.1 ± 3.0 years and 21 patients (19%) had a history of circumcision.

Mean operative time was 146 ± 23 min, scrotal skin was used to establish the neovagina in 14 patients (13%), and mean length of stay was 15 ± 3.4 days. All clinical and surgical characteristics are depicted in Table 1. Eighty five out of 108 patients were readmitted for the mostly cosmetical second step of gender reassignment. Twenty three patients canceled this, citing a subjectively adequate surgical outcome.

**Table 1:** Clinical and surgical characteristics of 108 transgender women undergoing penoscrotal flap inversion vaginoplasty between 2015 and 2018.

**Clinical characteristics**	
Age (years); mean (SD)	36 ± 13
ASA™ physical status (*n =* 106); mean (SD)	1.6 ± 0.6
Body mass index; mean (SD)	25 ± 6.1
Charlson Comorbidity Index; mean (SD)	0.3 ± 0.9
Duration of hormonal therapy (years; *n =* 92); mean (SD)	3.1 ± 3.0
Hx of circumcision; *n* (%)	21 (19)
Diabetes mellitus; *n* (%)	1 (0.93)
Hypertension; *n* (%)	11 (10)
Coronary heart disease; *n* (%)	1 (0.93)
Peripheral artery disease; *n* (%)	1 (0.93)
Chronic obstructive pulmonary disease; *n* (%)	2 (1.9)
Depression; *n* (%)	22 (20)
**Surgical characteristics**	
Operative time (min); mean (SD)	146 ± 23
Length of stay (days); mean (SD)	15 ± 3.4
Preoperative hemoglobin (mg/dl); mean (SD)	14 ± 0.9
Postoperative hemoglobin (mg/dl); mean (SD)	11 ± 1.1
Difference preoperative—postoperative hemoglobin (mg/dl); mean (SD)	−2.8 ± 1.1
Preoperative Qmax (ml/s; *n =* 98); mean (SD)	29 ± 10
Postoperative Qmax (ml/s; *n =* 34); mean (SD)	23 ± 11
Difference preoperative—postoperative Qmax (ml/s; *n =* 32); mean (SD)	−6.2 ± 11
Intraoperative use of scrotal skin; *n* (%)	14 (13)

*ASA, American Society of Anesthesiologists; SD, standard deviation*.

### Assessment of Short-Term Complications

A detailed summary of the number and proportion of all recorded complication types and grading according to the CDC is shown in Table 2. 44 ST complications were captured in 31 of 108 patients (29%; 95% confidence interval [CI] = 20–38%). Five patients (4.6%) suffered from rectal injury intraoperatively, of which one (0.93%) had to undergo protective colostomy. Wound infection (18%) and wound dehiscence (13%) were the most common postoperative complications. Four patients (3.7%) had a significant bleeding after surgery and required blood transfusions. There were two patients (1.9%; 95% CI = 0.22–6.5%) with a “major” ST complication (CDC grade IIIb), who required surgical re-intervention due to persistent postoperative bleeding (Table 2). Eleven patients (10%) developed more than one ST complication after PIV.

**Table 2:** Short- and long-term complications in 108 transgender women undergoing penoscrotal flap inversion vaginoplasty between 2015 and 2018.

	CDC^*^grading	**Management**	**Number of complications**	**Proportion, % (*n =* 108)**
**Short-term complications**. 44 complications in 31 patients (29%)				
**Intraoperative complications**				
Rectal injury	— ^*^	Protective colostomy (*n =* 1); surgical closure (*n =* 4)	5	4.6
**Postoperative short-term complications**				
Anemia/bleeding requiring transfusion	II	Blood transfusion	4	3.7
Postoperative bleeding	IIIb	Surgical revision	2	1.9
Wound infection	II	Antibiotics	19	18
Wound dehiscence	I	Conservative; clinical observation or diagnostic evaluation only, reinforced adhesive skin closure	14	13
**Long-term complications** 44 complications in 33 patients (31%)				
Urethral fistula.	IIIb	Surgical correction (2nd stage)	1	0.93
Urethral necrosis	IIIb	Surgical correction (2nd stage)	5	4.6
Meatal stenosis	IIIb	Surgical correction (2nd stage)	9	8.3
Neovaginal introitus stenosis	IIIb	Surgical correction (2nd stage)	10	9.3
Neovaginal atrophy	IIIb	Surgical correction (2nd stage)	15	14
Neoclitoral necrosis	IIIb	Surgical correction (2nd stage)	4	3.7

*CDC, Clavien-Dindo Classification*.

* *Not applicable to intraoperative complications*.

### Assessment of Long-Term Complications

Forty four LT complications within the first 6 months following two-staged PIV were captured in 33 of 108 patients (31%; 95% CI = 22-40%; Table 2). The most common complications were neovaginal introitus stenosis (9.3%), neovaginal atrophy (14%), and meatal stenosis (8.3%). None of the patients with intraoperative rectal injury presented with recto-vaginal fistula at LT follow-up. 10 patients (9.3%) developed more than one LT complication after PIV (Table 2).

### Evaluation of the CCI® and Multivariable Linear Regression Analysis

The mean CCI® of the overall cohort taking into account all recorded complications was 9.1 ± 12. When dichotomizing the cohort according to the sequence of performed procedures, the mean CCI® significantly decreased from 12 ± 14 (procedures 1–54) to 6.4 ± 9.8 (procedures 55–108; *p* = 0.021).

Age at time of surgery, the use of scrotal skin and number of surgeries performed were all significantly associated with the level of CCI® (R^2^ = 0.13). Duration of hormone therapy (*p* = 0.90), BMI (*p* = 0.98) and CI (*p* = 0.29) did not significantly influence the CCI®.

### Assessment of Functional Outcomes

Of 94 patients with available data at 6 months, 79 (84%) had neovaginal depth of ≥ 135 mm. Of 58 patients with data on self-reported orgasmic function at 6 months, 37 (64%) reported to be able to reach an orgasm. 19% reported pain. Overall sensibility was observed in 98% of the patients.

### Learning-Curve Analysis

**Figure 1:**
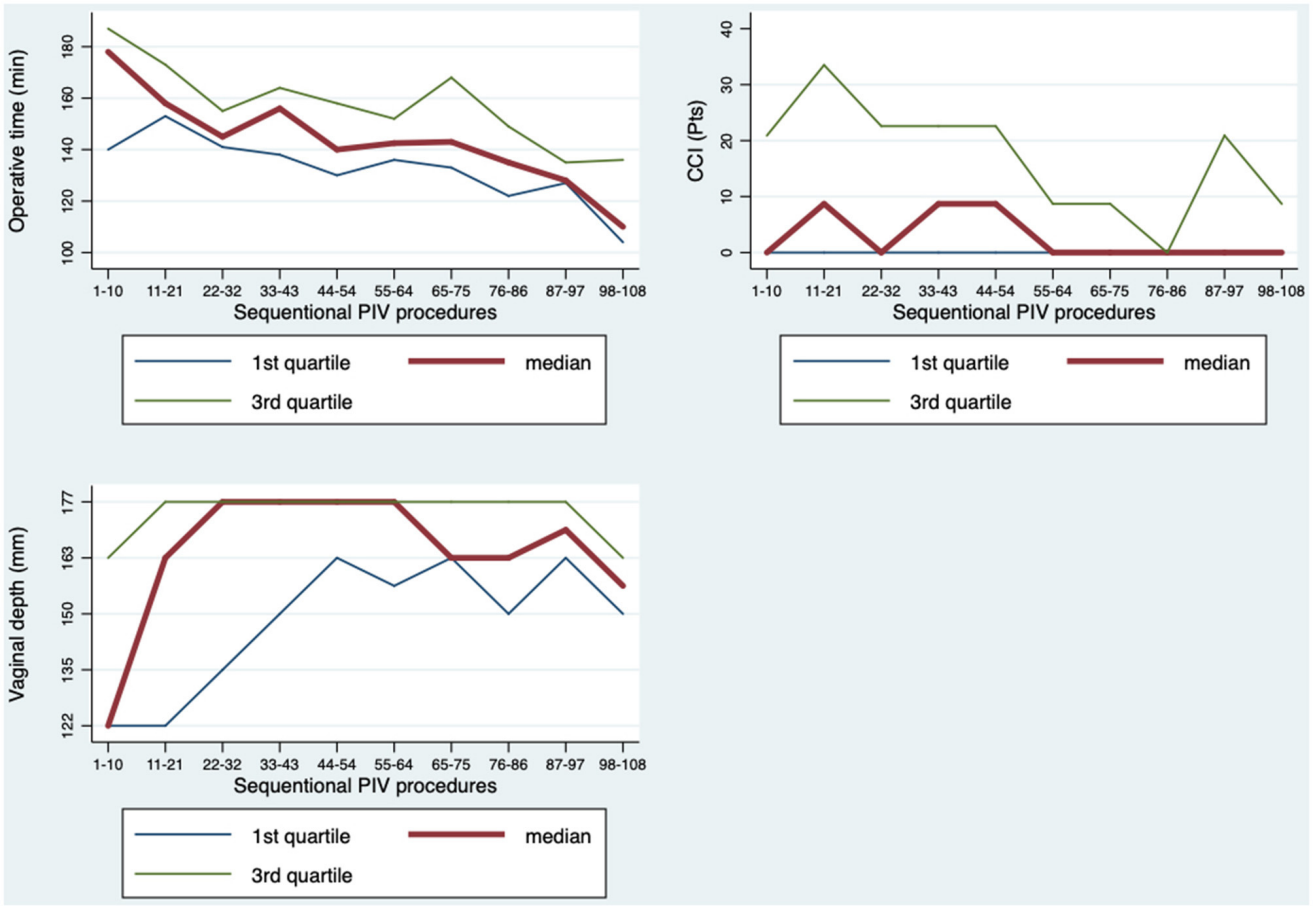
Learning curve analysis based on three factors: operating time, vaginal depth and complications.

We focused on operating time, vaginal depth and complications in 108 consecutive surgeries from one single surgeon. In a linear regression model a significant drop in operating time was observed (Figure 1) after ~22 surgeries. After a flattening of the standard curve, a second significant drop was observed after 76 surgeries with the tendency of further economization. The regression analysis showed a linear significance of *p* = 0.001 with adjusted R2 = 0.34.

The vaginal depth shows an initial increase followed by a stabilization between level 4–5 (163–177 mm) after about 22–32 cases (Figure 1).

The CCI® was applied in order to evaluate ST complications over time (Figure 1). After 54 surgeries, a significant drop of the CCI® was seen in the two-sided t-test comparing only the first and second half surgeries performed and second half surgeries performed (*p* = 0.021). This was also confirmed by a linear regression model, corrected for age (*p* = 0.034).

**Figure 2:**
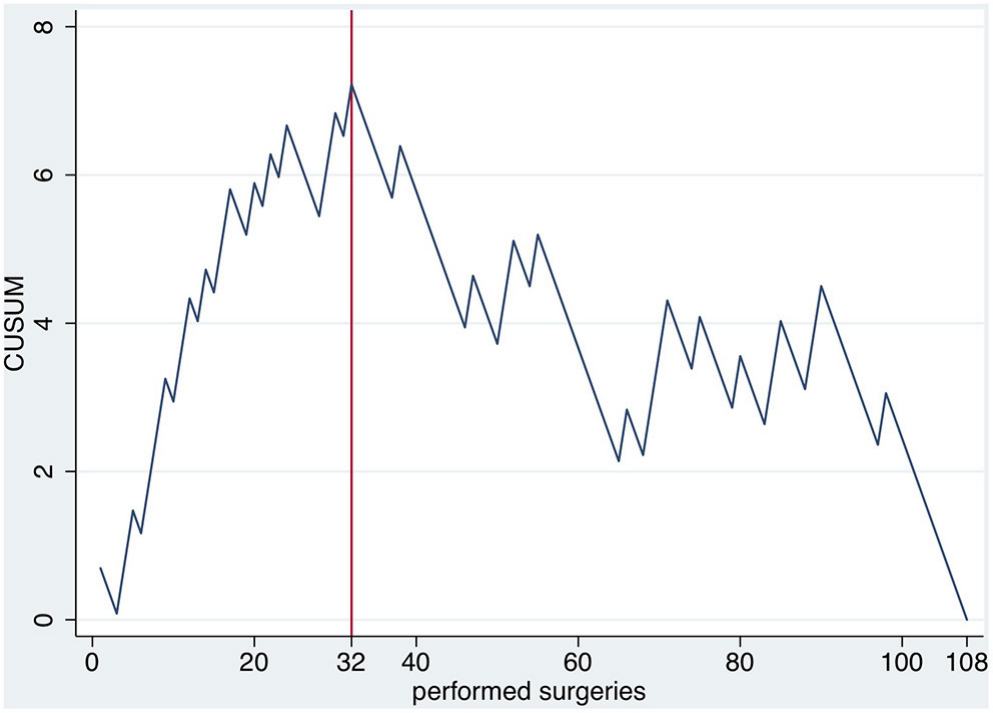
CUSUM of the occurrence of long term complications over time.

According to the CUSUM-analysis, LT complications reached a peak at 32 surgeries and declined thereafter, indicating a competence acquired by the surgeon (Figure 2).

## Discussion

In view of the rising numbers of GAS, analysis of complications, risk factors and learning-curve analysis of the PIV, which constitutes the most common surgical technique, is becoming increasingly important.

### Complications

In the current literature, postoperative ST complication rates of 28.7–32.1% at 3 months are described (8, 17, 18), which is in accordance with the rates observed in our study (30.6%). Moreover, male-to-female PIV surgery is described as a relatively safe operation technique with very few CDC high-grade complications (3, 8, 18). This is also mirrored by our results, with >90% being Clavien I and II complications. ST-complications requiring surgical revision were 6.5%, which is within the range of rates reported so far. The overall incidence of rectal injuries was low (4.6%) as reported in other studies (10, 11, 19, 20), all of which were repaired intraoperatively.

However, in contrast to the literature, our multiple regression analysis based on the more sensitive CCI® shows that age at the time of surgery is significantly associated with complications, as younger patients were more likely to have postoperative bleedings (8). This correlates with the surgeons' clinical and intraoperative experience, that younger patients have stiffer tissue and more pelvic floor muscles, which may in turn explain a more difficult anatomic preparation of the neovagina and consecutive bleedings. However, our multivariate analysis showed that the duration of hormone replacement therapy, which was significantly longer in older patients, does not significantly correlate with complications. In line with Gaither et al., the duration hormone replacement therapy, which is discussed to predispose for local, i.e., penile skin (8, 20) and systemic complications (21), are not associated with perioperative complications in our analysis. Despite the continuation of the hormone therapy at the time of surgery, no thromboembolic events were seen in our cohort.

In line with Goddard et al., patients with free-skin grafts developed significantly more complications, in particular vaginal stenosis. The finding of no significant differences in surgical outcome between PIV with or without the use of additional full-thickness skin graft (22) cannot be supported.

In line with Ives et al., BMI was no risk factor for complications in PIV and should therefore not preclude patients from GAS (23).

Regarding LT-complications, our rates are higher than described in the literature, e.g., neovaginal atrophy (13.7%, literature 1–12%) (9, 11, 20, 22, 24, 25) and clitoral necrosis (3.7%, literature 1–3%) (10, 11, 19, 20, 22, 24) urethral necrosis (4.6%, literature 0.6%) (10), or at the upper end of the range, e.g., introitus strictures (9.3%, literature 2.5–15%) (11, 19, 22) as well as urethral fistulas (0.9%, literature 0.6–1%) (10), Compared to other studies, it remains unclear, whether the latter included the first PIVs performed. Our surgeons LC biases the results, as complications become less frequent with increasing surgical expertise. Regarding meatal stenosis, our rates of 8.3% (literature 4–40%) are at the lower range, which may be explained by the late transurethral catheter removement on the 10th postoperative day, compared day 2–5 in other studies.

Regarding functional outcome, vaginal depth of ≥135 mm (average erect penis length in German man 14.5 cm) (26) was reached in 84% of the patients, which corresponds to the literature (9, 20, 25). The sexual function in terms of the ability to achieve orgasm was 64%, compared to 48–84% described for PIV in the literature (9, 20, 24, 25) clitoral sensitivity was 98% in our cohort. However, only 58 of 108 replied to the question and none of the patients had performed neovaginal intercourse 6 months postoperatively before the second stage of GAS. Further follow-up and an evaluation based on the oMtFSFI (operated Male-to-Female Sexual Function Index) (27) is required and is going be performed after validation in German, in order to sufficiently address this aspect.

### Learning-Curve

LC analysis is a crucial aspect in surgeons' education and—when applied correctly—increases patient safety as it allows to define the training needed in order to achieve a high-quality level of surgical performance (28, 29).

So far there have only been two studies focusing on the LC of PIV, based primarily on operative time and vaginal depth as outcome parameters *via* scatterplot analysis and the statistical comparison of arbitrary splitting groups (12, 13).

The present study with 108 patients is the biggest LC-analysis of PIV. Based on internationally recognized standards, three aspects were chosen for performance analysis: operating time, functional outcome and complications.

A significant reduction in operating time is seen after 22 surgeries, which is then followed by a plateau and a further economization after 76 surgeries. These results are comparable to Falcone et al., who described a learning plateau after about 30 cases. Regarding vaginal depth, a similar number of surgeries is required. However, these two factors alone do not sufficiently reflect the quality of the PIV performance. The central column is the CUSUM analysis of complications. CUSUM shows a peak of cumulative complications after 32 surgeries and declines thereafter. The advantage of CUSUM is the possibility to specifically evaluate a surgeon's performance over time. Our analysis supports the finding, that about 30 surgeries are required to overcome the learning-curve.

### General Limitations

A limitation of the study is the average follow-up time of 6.3 months. Long-term complications that develop after this time are therefore not included. An extended FU is required to further support the results. Our learning-curve data is based on a single surgeon's experience, only. In order to analyze the significance of surgical experience, a comparative analysis with a less experienced surgeon ought to be performed. Finally, with respect to sexual function outcome, a more sophisticated analysis based on the oMtFSFI is necessary.

## Conclusions

The PIV is a well-established technique in GAS, associated with minor complications leading to low rates of ST-revision surgery. Younger age has a significant impact on complications. The length of hormonal therapy, a high BMI or a history of circumcision has no impact on complications.

Learning-curve analysis of an experienced surgeon shows a significant reduction in operation time and complications as well as good functional outcomes after about 32 surgeries.

## Data Availability Statement

The original contributions presented in the study are included in the article/supplementary materials, further inquiries can be directed to the corresponding author/s.

## Ethics Statement

Ethical review and approval was not required for the study on human participants, in accordance with the local legislation and institutional requirements.

## Author Contributions

SR: protocol/project development, data acquisition, and manuscript editing. RD, MF, CM, AS, PM, SW, and PG: manuscript editing. MV and TL: data analysis and manuscript editing. IF: data acquisition. MH: protocol/project development, data analysis, and manuscript editing. VM: protocol/project development and manuscript writing. All authors contributed to the article and approved the submitted version.

## Conflict of Interest

The authors declare that the research was conducted in the absence of any commercial or financial relationships that could be construed as a potential conflict of interest.

## Publisher's Note

All claims expressed in this article are solely those of the authors and do not necessarily represent those of their affiliated organizations, or those of the publisher, the editors and the reviewers. Any product that may be evaluated in this article, or claim that may be made by its manufacturer, is not guaranteed or endorsed by the publisher.

## Abbreviations

CI: Charlson index
CDC: Clavien Dindo Classification
CCI®: Comprehensive Complications Index
CUSUM: Cumulative sum
FU: follow-up
GD: gender dysphoria
GAS: gender-affirmation surgery
LC: learning-curve
LT: long-term
MTF: male-to-female
oMtFSFI: operated Male-to-Female Sexual Function Index
PIV: penile-inversion vaginoplasty
ST: short-term
SD: standard deviation
WPATH: World Professional Association of Transgender Health.
